# Therapeutic hypothermia increases the risk of cardiac arrhythmia for perinatal hypoxic ischaemic encephalopathy: A meta-analysis

**DOI:** 10.1371/journal.pone.0173006

**Published:** 2017-03-08

**Authors:** Wei Zhang, Meizhu Lu, Chenlong Zhang, Ruwen Zhang, Xiaofeng Ou, Jianju Zhou, Yan Li, Yan Kang

**Affiliations:** 1 Department of Critical Care Medicine, Sichuan University West China Medical Center, Chengdu, Sichuan, China; 2 Department of Critical Care Medicine, First People's Hospital of Zunyi in Guizhou Province, Third Clinical Hospital of Zunyi Medical College, Zunyi, Guizhou, China; 3 Department of Neurology, People’s Hospital of Yishui County, Yishui, Shandong, China; Azienda Ospedaliero Universitaria Careggi, ITALY

## Abstract

**Objective:**

To determine whether therapeutic hypothermia after hypoxic ischaemic encephalopathy (HIE) in neonates increases the risk of cardiac arrhythmia during intervention.

**Design:**

A meta-analysis was conducted using a fixed-effect model. Risk ratios, risk differences, and 95% confidence intervals, were measured.

**Data sources:**

Studies identified from the Cochrane Central Register of Controlled Trials, MEDLINE, EMBASE, Google Scholar, previous reviews, and abstracts from onset to August, 2016.

**Review methods:**

Reports that compared therapeutic hypothermia with normal care for neonates with HIE and that included data on safety or cardiac arrhythmia, which is of interest to patients and clinicians, were selected.

**Results:**

We found seven trials, encompassing 1322 infants that included information on safety or cardiac arrhythmia during intervention. Therapeutic hypothermia considerably increased the combined rate of cardiac arrhythmia in the seven trials (risk ratio 2.42, 95% confidence interval 1.23 to 4.76. p = 0.01; risk difference 0.02, 95% CI 0.01 to 0.04) during intervention.

**Conclusions:**

In infants with hypoxic ischaemic encephalopathy, therapeutic hypothermia is associated with a consistent increase in cardiac arrhythmia during intervention.

## Introduction

Perinatal hypoxic ischaemic encephalopathy is the second leading cause of neonatal death, and it also globally accounts for the third major cause of childhood death [1]. Studies showed that neural damage after hypoxic ischaemia is delayed in response to several hours of acute hypoxic ischaemia [2], and therapeutic hypothermia can be used in newborn infants with HIE to reduce the risk of death and neurological impairments [3]. Currently, therapeutic hypothermia is the standard clinical care for moderate-to-severe perinatal HIE in high-income countries [4]. Therefore, previous studies have focused on the efficacy of therapeutic hypothermia in infants with HIE, while adverse effects or complication data might be ignored by clinicians.

Among these unfavourable effects, cardiac arrhythmia is one of the most common complications during the intervention or in the hospital. Several clinical trials [5–11] reported some or all of the adverse effects, but the results of these trials are not conclusive. An updated systematic review issued by the Cochrane Library in 2013 reported that 72 hours of moderate hypothermia started within 6 hours of birth has a negative effect on cardiac arrhythmia during intervention or in the hospital [12] Thus, cardiac arrhythmia adverse events remain ambiguous and controversial when hypothermia is used in a patient with HIE.

We aimed to perform a meta-analysis of relative studies and recently available data from published studies that defines how much therapeutic hypothermia might increase the risk of cardiac arrhythmia after hypoxic ischaemia. We will also determine its adverse effects during intervention in infants who survive perinatal HIE, and consider whether the therapeutic measure of hypothermia is more carefully applied to infants with moderate-to-severe encephalopathy. In the future, we will search for precautions to reduce the risk of cardiac arrhythmia according to the outcomes of our study.

## Materials and methods

### Search methods

Relevant studies were identified from MEDLINE (using the Mesh terms (“infant, newborn” AND “asphyxia and hypoxic ischemic encephalopathy”) and “hypothermia”), EMBASE, and the Cochrane Central Register of Controlled Trials, previous reviews including abstracts, cross-references, conferences, expert informants, symposia proceedings, and via searching journals by hand. We updated this search in August, 2016, and did not include the additional unpublished data and unpublished studies. Studies were identified by our group of authors, and relevant studies were selected by consensus.

### Selection criteria

We included all randomised controlled studies comparing the use of therapeutic hypothermia with standard care (normothermia) for perinatal HIE. The methodological quality of the studies was assessed, we were advised by the Cochrane Collaboration neonatal review group, which relied on the use of randomisation and blinding to the intervention, and which was also based on completeness and blinding of follow-up [13]. The study participants met the following criteria: 1. Newborn infants, 36 weeks gestation or greater; 2. Evidence of birth asphyxia, with each enrolled infant satisfying at least one of the following criteria: (i) Apgar score of 5 or less at 10 minutes; (ii) mechanical ventilation or resuscitation at 10 minutes; (iii) cord pH less than 7.1, or an arterial pH less than 7.1, or base deficit of 12 or more within 60 minutes of birth; 3. Evidence of encephalopathy according to the Sarnat staging (Sarnat 1976; Finer 1981) as follows: Stage 1 (mild): hyper-reflexia, hyper-alertness, dilated pupils, hypotonia with weak suck, Moro and seizures; Stage 2 (moderate): hyper-reflexia, lethargy, bradycardia, miosis, hypotonia with weak suck, miosis and seizures; Stage 3 (severe): flaccidity, stupor, decreased stretch reflexes, small to mid-position pupils that react poorly to light, hypothermia and absent Moro; 4. No major congenital abnormalities recognisable at birth. The requirement for intervention is determined when the following criteria are met: 1. Therapeutic hypothermia (whole body and/or selective cooling) initiated prior to 6 hours after birth versus no cooling (normothermia); 2. Cooling duration ranges from 48 to 72 hours or greater; and 3. Cooled body temperature ranges from 33 to 35°C (rectal temperature).

### Data collection and analysis

In our studies, cardiac arrhythmia was regarded as the adverse event assessment primary outcome in infants with HIE. The follow-up time window for cardiac arrhythmia adverse effects was identified as during intervention or while the patient remained in the hospital (in-hospital). We selected reports that included data to analyse the effect of hypothermia on the rate of cardiac arrhythmia during intervention or in-hospital.

### Statistical analysis

Meta-analysis was performed with the Review Manager software (RevMan Version 5.3; Nordic Cochrane Centre, Copenhagen, Denmark) using the Mantel-Haenszel method and a fixed-effect model. We calculated risk ratios (RR) and risk difference (RD) for dichotomous variables, with 95% confidence intervals (CI), which is the reciprocal of the risk difference, with 95% confidence intervals. Encephalopathy grade rated by amplitude integrated electroencephalography was considered to be equivalent to that of the clinical assessment [3]. All statistical tests were two-sided and analyses were not corrected for multiple comparisons. A P value <0.05 was considered statistically significant. We examined heterogeneity among studies using the *χ*^2^ and the *I*^2^ tests.

## Results

**Fig 1:**
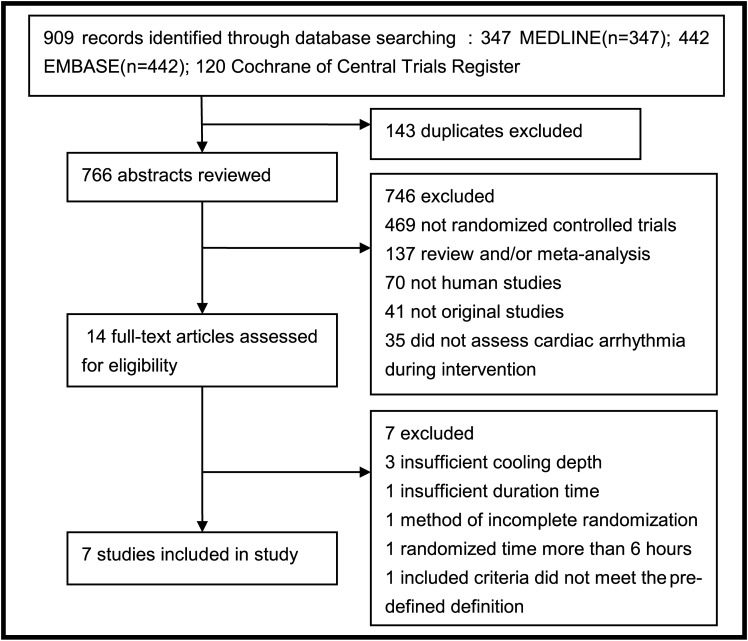
Flow-chart of study selection process.

Our literature search identified 909 unique records (Fig 1). We compiled a dataset of 1322 infants with HIE from 7 randomised controlled trials, which at least included cardiac arrhythmia data [5–11]. A summary of the trial characteristics is shown in Tables 1 and 2. These trials had similar inclusion criteria: evidence of birth asphyxia, at least 36 weeks gestation at recruitment, random allocation was completed by 6 hours after birth, a target rectal temperature range from 33.0~35.0°C, and temperature was maintained within that range for 48~72 hours. Cooling was induced using cooling blankets placed under the infants and/or achieved by circulating cooling fluid in a cap (systematic and/or selective cooling).

**Table 1:** Baseline characteristics of the included trials on therapeutic hypothermia vs. standard care for perinatal hypoxic ischaemic encephalopathy.

Source	Main Inclusion	Sample Size	Age at randomization, h		Birth Weight, g		Male, %		Umbilical Artery PH		Base Excess		Apgar (5',10'*)#	
			Cooling	Control	Cooling	Control	Cooling	Control	Cooling	Control	Cooling	Control	Cooling	Control
Shankaran,2005	Apgar score ≤5(10')	208	4.3	4.3	3385	3370	51 (50)	67 (63)	6.9	6.8	-18.5	19.9	NR	NR
Gluckman,2005	Apgar score ≤5(10')	334	NR	NR	3399	3504	64(55.2)	58(49.2)	6.9	6.9	-21.0	-20.4	NR	NR
Azzopardi,2009	Apgar score ≤5(10')	325	4.7	4.7	3450	3350	101(62)	88(54)	NR	NR	NR	NR	4	4
Zhou,2010	Apgar score≤5(5')	194	4.1	4.0	3360	3299	87(87)	78(83)	NR	NR	NR	NR	NR	NR
Simbruner,2010	Apgar score ≤5(10')	125	4.6	4.1	3300	3300	31(50.0)	33(52.4)	6.9	6.9	-19.4	-19.5	3.4	3.2
Bharadwaj,2012	Apgar score ≤6(10')	124	NR	NR	2967	2899	43(69)	37(60)	7.09	7.08	-17.6	-17.6	5.34*	5.26*
Joy,2013	Apgar score ≤5(10')	118	NR	NR	2840	2910	NR	NR	7.06	7.09	-19.4	-19.1	3.81*	3.45*

*, at 10 minutes;

^#^, 5 minutes, 10 minutes;

NR, not report

**Table 2:** Baseline characteristics of the included trials on therapeutic hypothermia vs. standard care for perinatal hypoxic ischaemic encephalopathy.

Source	Primary outcome	Follow-up Period	Ratio cooled: controls	Cooling Method	Core Temperature achieved, °C	Duration of Cooling, h	Gestational age, wk		Duration of hospital stay, d		Moderate encephalopathy -n (%)		Severe encephalopathy -n (%)	
							Cooled	Control	Cooled	Control	Cooled	Control	Cooled	Contol
Shankaran,2005	Rates of death, moderate and severe disability	18 months	102:106	Systemic	33.5	72	NR	NR	19.9	20.9	69 (68)	66 (62)	32 (32)	40 (38)
Gluckman,2005	Rates of death and severe disability	18 months	116:118	Selective	34–35	72	38.9	39.1	NR	NR	63 (54)	76 (64)	42 (36)	32 (27)
Azzopardi,2009	Rates of death and severe disability	18 months	163:162	Systemic	33–34	72	40.3	40.1	NR	NR	65 (40)	67 (41)	98 (60)	95 (59)
Zhou,2010	Rates of death and severe disability	18 months	100:94	Selective	34.5–35	72	NR	NR	NR	NR	41 (41)	41 (44)	38 (38)	35 (37)
Simbruner,2010	Rates of death and severe disability	18 months	64:65	Systemic	33–34	72	39.2	39.4	18.4	29.9	24 (38.7)	17 (27)	38 (61.3)	46 (73)
Bharadwaj,2012	Rate of death and neurodevelopmental outcome	6 months	62:62	Systemic	33–34	72	39.9	40.0	7.68	8.2	55 (88.7)	54 (87)	7 (11.3)	8 (13)
Joy,2013	Oxidative stress and neurological outcome	Discharge	58:58	Systemic	33–34	72	NR	NR	NR	NR	49 (84.5)	51 (87.9)	9 (15.5)	7 (12.1)

NR, not report

Seven randomised controlled trials were excluded for insufficient cooling depth [14–16], insufficient duration time [17], method of incomplete randomisation [18], randomised time more than 6 hours (up to 10 hours) [19], and study included criteria that do not meet the pre-defined definition of peripartum asphyxia [20].

**Fig 2:**
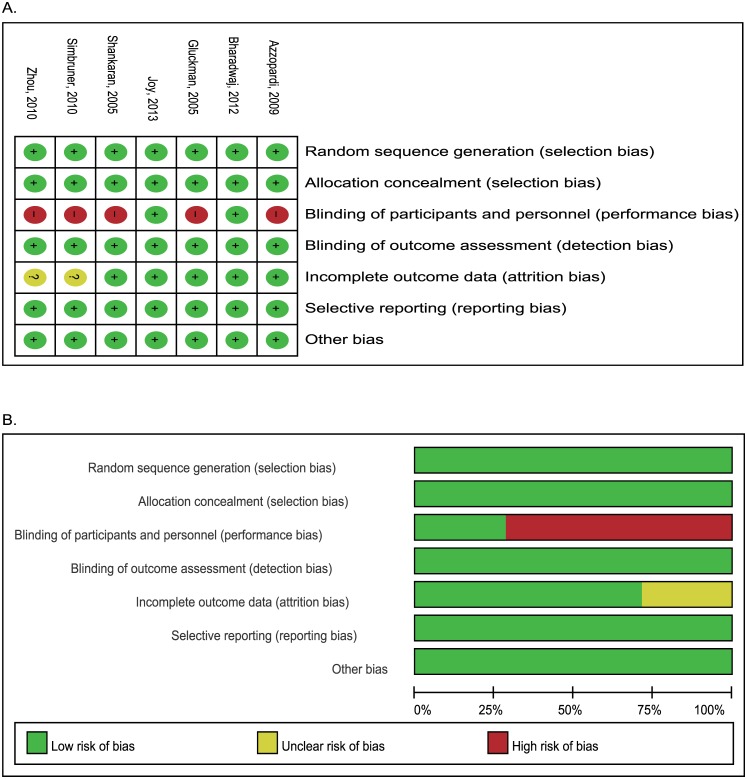
Risk bias of summary (A) and graph (B) according to review authors’ judgement.

**Fig 3:**
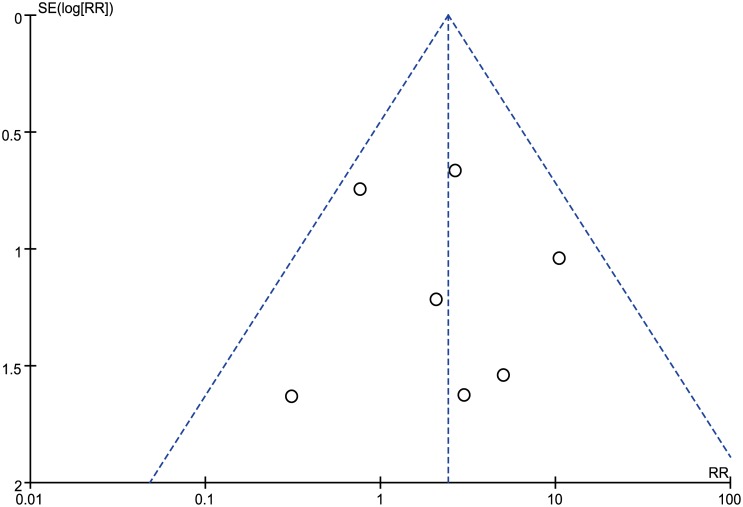
Funnel plot of included randomized controlled trials.

Of the seven trials that reported the rate of cardiac arrhythmia during intervention, five had caregivers who were not blinded to the treatment assignment (Fig 2) [5–9]. The publication bias funnel plot for cardiac arrhythmia during intervention or in-hospital is shown in Fig 3. The methodological quality of the trials included in the assessment of cardiac arrhythmia was reported in a 2013 update Cochrane review [12]. The two remaining studies used an appropriate methodology.

**Fig 4:**
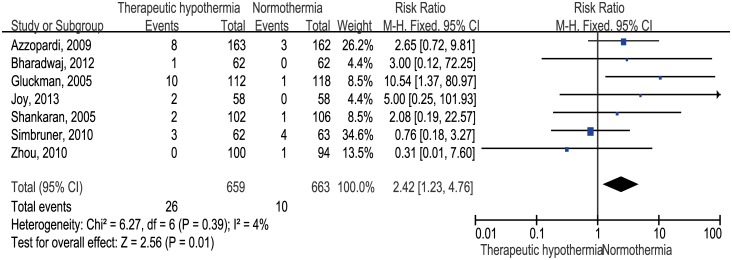
Therapeutic hypothermia versus normothermia: Analysis by cardiac arrhythmia ("events") during intervention.

We identified seven trials including 1322 infants with HIE, and these trials contained information about cardiac arrhythmia during intervention. Therapeutic hypothermia significantly increased the rate of cardiac arrhythmia (risk ratio 2.42, 95% confidence interval 1.23 to 4.76, p = 0.01; risk difference 0.02, 95% CI 0.01 to 0.04) during intervention (Fig 4).

## Discussion

Hypothermia is among the most severe complications in critically ill patients with severe shock and multiple traumas, which comprises *the death triangle* when combined with coagulopathy and acidosis. Hypothermia is also widely used clinically because moderate hypothermia was shown to reduce brain metabolism and alleviate brain injury, and thus, it plays a protective role in encephalopathy or brain injury. However, in clinical, cardiac arrhythmia induced by therapeutic hypothermia in infants with HIE may be underestimated because of its low morbidity. In the above-mentioned studies, the focus was mostly on mortality and neurological outcomes, while there were few reports on adverse effects of cardiac arrhythmia. In some meta-analyses, there were no reports of cardiac arrhythmia in infants with HIE [21]. In this study, we confirmed that therapeutic hypothermia increased the risk of cardiac arrhythmia in infants with HIE during the intervention. Recently, therapeutic hypothermia was shown to be ineffective, and even harmful, in children with out-of-hospital cardiac arrest [22] and in adults with traumatic brain injury [23] and infection/inflammation [24]. Thus, we investigated the undesirable outcomes that are involved in the adverse effects or complications of therapeutic hypothermia.

Our research has several advantages. First, we have a well-defined goal so we selected only a single variable for performing the meta-analysis, to preclude confounding factors from other variables. Second, the variable of cardiac arrhythmia is easy to measure during the intervention in infants with HIE, which reduces the risk of dropout. Third, the outcome of our research is consistent with the advances in the present studies, which show that therapeutic hypothermia in infants with HIE should be taken into consideration by clinicians.

### Limitations of study

Although moderate therapeutic hypothermia was shown to increase the risk of cardiac arrhythmia in infants with moderate-to-severe HIE compared with standard care, there were still some limitations. In the included seven randomised controlled trials, the rate of cardiac arrhythmia was reported, but it was not the primary outcome. Whereas, in our study, we regarded it as the primary outcome.

The sample size might still be restricted in a poor level due to the episodes were reported in few literatures. Therefore, in order to validate our conclusions, we need to design some high-quality clinical trials with a safety assessment that includes cardiac arrhythmia in patients undergoing moderate therapeutic hypothermia. This will help to search for the relevant precautions to prevent the adverse effect of cardiac arrhythmia.

Finally, there were lack of recent studies due to the clarified efficacy of reduction mortality and improving neurological outcomes on therapeutic hypothermia in infants with HIE. Moreover, cardiac arrhythmia is usually regarded as a secondary outcome in some studies, so that there are few studies related to this topic.

Although the morbidity is low on therapeutic hypothermia induced cardiac arrhythmia in infants with HIE, actually, the problem is still deserved to be focused by clinicians. Because the conclusions may provide a direction on medical study of pathobiological mechanism of hypothermia induced cardiac arrhythmia.

## Conclusions

In infants with HIE, therapeutic hypothermia increases the risk of cardiac arrhythmia during intervention or in-hospital.

## Supporting information

S1 Table PRISMA 2009 checklist. (DOC)

S1 Fig PRISMA 2009 flow diagram. (EPS)
